# Prevalence and Determinants of General and Central Obesity in Central-Southern Bulgaria: Associations with Cardiometabolic Risk and Lifestyle Factors

**DOI:** 10.3390/healthcare14091126

**Published:** 2026-04-22

**Authors:** Steliyana Valeva, Nazife Bekir, Katya Mollova, Andriana Kozareva, Ivelina Stoyanova, Pavlina Teneva

**Affiliations:** Department of Health Care, Medical College, Trakia University, Armeiska Street 9, 6000 Stara Zagora, Bulgaria; nazife.bekir@trakia-uni.bg (N.B.); katya.mollova@trakia-uni.bg (K.M.); andriana.kozareva@trakia-uni.bg (A.K.); ivelina.stoyanova@trakia-uni.bg (I.S.); pavlina.teneva@trakia-uni.bg (P.T.)

**Keywords:** obesity, central obesity, cardiometabolic risk, lifestyle factors, age-related changes

## Abstract

**Highlights:**

**What are the main findings?**

- General and central obesity are highly prevalent (68.4% and 66.9%), with nearly one-quarter of normal-weight individuals exhibiting high-risk central adiposity.
- While BMI remains a robust predictor, WHtR increases progressively with age, even when BMI stabilizes, reflecting age-related visceral fat redistribution.

**What are the implications of the main findings?**

- Reliance on BMI alone may underestimate cardiometabolic risk; the integration of WHtR improves identification of “hidden” risk in aging and normal-weight individuals.
- Simple, cost-effective anthropometric tools such as WHtR provide a reliable basis for risk stratification in primary healthcare and comparable urban populations.

**Abstract:**

**Background:** Obesity represents a major public health challenge worldwide and contributes substantially to the burden of type 2 diabetes and hypertension. While body mass index (BMI) is widely used in clinical practice, indices reflecting central adiposity may provide additional prognostic value. This study aimed to assess the prevalence of general and central obesity in an adult population across different age groups from Stara Zagora, Bulgaria, and to examine their associations with cardiometabolic outcomes and lifestyle factors. **Methods:** A quasi-representative cross-sectional study was conducted among 3512 adults (mean age 53.7 ± 14.9 years). Anthropometric indices, including BMI, waist circumference, waist-to-hip ratio, and waist-to-height ratio were measured. Cardiometabolic outcomes included diabetes, hypertension, and their combined presence. Multicollinearity was assessed using the Variance Inflation Factor (VIF), and the discriminatory ability of indices was evaluated using Receiver Operating Characteristic (ROC) analysis and DeLong’s test. **Results:** The prevalence of overweight/obesity (BMI ≥25) was 68.4%, while central obesity (WHtR ≥0.5) affected 66.9% of participants. BMI demonstrated the highest discriminatory ability in this dataset for hypertension (AUC = 0.852) and diabetes (AUC = 0.796), significantly outperforming WC and WHR (*p* < 0.05). However, 24.4% of individuals with normal BMI exhibited high-risk central adiposity. Significant sex-specific differences were observed: short sleep duration (<6 h) was a strong predictor of obesity in women (aOR = 2.98), whereas smoking showed stronger associations in men. Age-stratified analyses revealed that while BMI stabilizes in the oldest age group (75–89 years), WHtR continues to increase, reflecting age-related redistribution of visceral fat. A strong protective effect of physical activity was observed, supported by quasi-complete separation in active subgroups. **Conclusions:** General and central obesity represent a substantial health burden in this urban population. While BMI remains a robust screening tool, the integration of WHtR enhances the identification of “hidden” cardiometabolic risk particularly in older adults and individuals with normal BMI. Given the quasi-representative nature of the sample, these findings are primarily generalizable to similar urban populations and may inform targeted regional public health strategies.

## 1. Introduction

Obesity is a chronic multifactorial condition that poses a major burden for healthcare systems worldwide. It is strongly associated with type 2 diabetes, arterial hypertension, cardiovascular disease, and increased healthcare utilization [1]. Over the past decades, its global prevalence has risen steadily, reaching epidemic proportions and establishing obesity as one of the most significant modifiable risk factors for non-communicable diseases [2]. According to recent estimates, more than 1 billion adults worldwide are living with obesity, with projections indicating a continued upward trend in both developed and developing countries [3]. Beyond individual morbidity, the clinical and economic consequences of obesity place sustained pressure on primary care systems and long-term disease management.

In Bulgaria, national and regional data from Sofia and Plovdiv indicate a substantial prevalence of overweight and obesity, consistent with broader European trends [4,5,6]. However, most available data remain descriptive and are largely based on body mass index (BMI), which does not account for fat distribution [4,5]. Given the heterogeneity of cardiometabolic risk among individuals with similar BMI values, reliance on BMI alone may lead to underestimation of high-risk individuals in clinical practice.

A critical challenge in contemporary clinical assessment is the phenomenon of sarcopenic obesity, particularly prevalent in aging populations. With advancing age, the progressive decline in lean muscle mass is often accompanied by an increase in visceral adipose tissue, even in the absence of significant changes in total body weight [7]. This internal redistribution of fat renders BMI an increasingly insensitive marker of metabolic risk in older adults. Consequently, individuals classified as having normal weight may still present with elevated cardiometabolic risk, contributing to the so-called “obesity paradox.” This age-related shift in body composition underscores the need for anthropometric indicators that capture fat distribution rather than total body mass, particularly in older populations where risk may otherwise remain undetected [8].

Central obesity, commonly assessed through waist circumference (WC), waist-to-hip ratio (WHR), and waist-to-height ratio (WHtR), has been increasingly recognized as a more reliable predictor of cardiometabolic risk than BMI alone [9,10]. Visceral adiposity is metabolically active and contributes to insulin resistance, chronic low-grade inflammation, and sympathetic nervous system activation—mechanisms closely linked to the development of diabetes and hypertension [11,12,13]. While BMI remains a useful proxy for overall adiposity, indices incorporating waist measurements, particularly WHtR, provide superior insight into fat distribution and its metabolic consequences [14]. WHtR has gained particular attention due to its simplicity, applicability across age groups, and the use of a universal threshold (≥0.5), which facilitates its implementation in primary healthcare settings [15,16].

Despite the growing body of evidence supporting the clinical relevance of central obesity indicators, region-specific analytical data integrating multiple anthropometric indices with cardiometabolic outcomes remain limited in Eastern Europe. In particular, there is a lack of studies combining multivariable modeling with comparative evaluation of the discriminative performance of different indices, especially in urban populations, where environmental and behavioral factors significantly influence risk profiles. Furthermore, age-related patterns of central obesity and their interaction with lifestyle factors remain insufficiently explored in the Bulgarian context.

Post-COVID-19 pandemic data from Eastern Europe indicate a marked increase in sedentary behavior and screen-based occupational patterns, contributing to the rising prevalence of abdominal obesity in urban populations [17]. In countries such as Bulgaria, the rapid transition toward more automated and sedentary work environments in industrialized regions, including Stara Zagora, has created a “pro-obesogenic” environment. At the same time, traditional dietary patterns are increasingly being replaced by the consumption of highly processed foods, further weakening the association between BMI and actual metabolic health.

Stara Zagora represents a demographically mixed and industrialized urban region with an aging population—factors that may contribute to an increased burden of central obesity and cardiometabolic risk compared to national averages. By examining a large, approximately representative urban cohort, the present study aims to provide region-specific evidence aligned with established anthropometric standards and clinical practice.

To date, no comprehensive screening data have been published for this region, and the burden of central obesity remains insufficiently characterized. Therefore, the present study aimed to assess the prevalence of general and central obesity in the adult population across different age groups of Stara Zagora, Bulgaria, to investigate their associations with diabetes, hypertension, and behavioral factors, and to evaluate the discriminative capacity of different anthropometric indices using receiver operating characteristic (ROC) analysis and multivariable logistic regression models. By integrating prevalence estimates with analytical modeling, this study seeks to support improved risk stratification and screening strategies in comparable urban populations.

Given the quasi-representative nature of the sample, the findings should be interpreted as applicable primarily to similar urban populations rather than at a national level.

## 2. Materials and Methods

### 2.1. Study Design

A cross-sectional, population-based observational study was conducted among a representative sample of the adult population across different age groups of the city of Stara Zagora, Bulgaria. The primary aim was to assess the prevalence of overweight, general, and central obesity, and to examine their associations with demographic and behavioral factors.

The study was carried out between May 2024 and November 2025 at the Medical College of Trakia University, Stara Zagora, within the framework of project No. MK/2024 “Development of an automated system for screening studies in public health”. The protocol was conducted in accordance with the Declaration of Helsinki and approved by the Ethics Committee of Trakia University (Protocol No. 30; approval date: 25 April 2024).

### 2.2. Population and Inclusion/Exclusion Criteria

**Figure 1:**
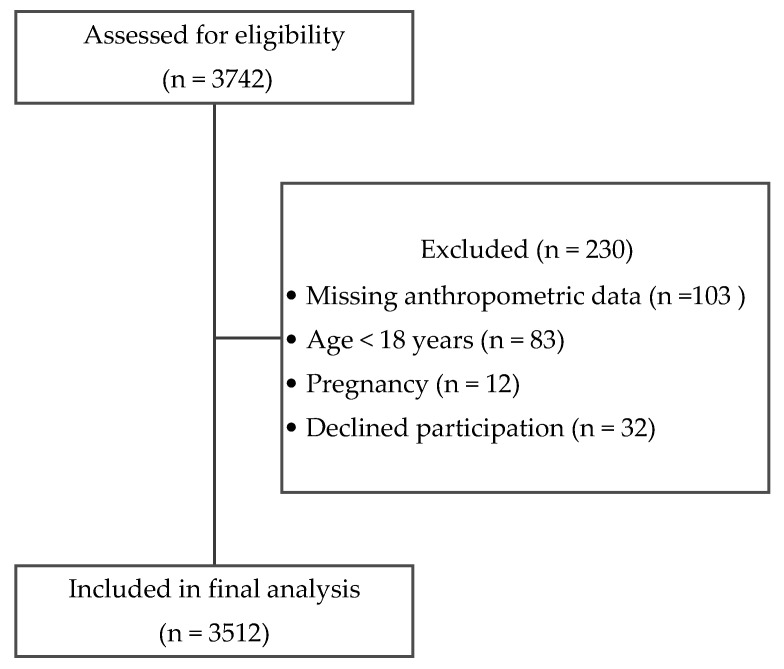
Flow diagram of participant recruitment and inclusion.

A total of N = 3512 participants (men and women) aged ≥18 years were included (Figure 1).

The required sample size was calculated using Cochran’s formula:

n = (Z^2^ × p × (1 − p))/e^2^, assuming p = 0.5, a 95% confidence level (Z = 1.96), and a margin of error of 2% (e = 0.02). For a population of approximately 135,000, the minimum required sample size was 2359. The final sample exceeded this requirement. Given the achieved sample size, the study provides high statistical precision for prevalence estimates, with a margin of error below ± 2% at the 95% confidence level. In addition, the sample size ensures adequate statistical power for detecting even small effect sizes.

Participants were recruited through community-based sampling from primary care facilities, workplaces, and public locations within the urban area. Although probability-based sampling was not feasible, efforts were made to ensure diversity in age, sex, and occupational background.

The sampling approach should be considered non-random and convenience-based, which may limit generalizability. In particular, certain population subgroups (e.g., individuals less engaged with healthcare services or with different socioeconomic profiles) may be underrepresented, potentially affecting external validity.

Inclusion criteria were: age ≥18 years; permanent residence in Stara Zagora; provision of written informed consent; and absence of acute illness at the time of assessment. Exclusion criteria included: age <18 years; pregnancy; terminal-stage chronic disease; and missing key anthropometric data.

All participants received oral and written information about the study objectives and potential benefits and risks. They signed an informed consent, guaranteeing the confidentiality of personal data and the right to withdraw from participation at any stage without negative consequences.

### 2.3. Research Methods

#### 2.3.1. Demographic and Behavioral Data

Data on age and sex were collected. Behavioral variables, including smoking status, sleep duration, presence of chronic diseases, and physical activity, were assessed using a self-reported questionnaire, which may be subject to recall and reporting bias.

Physical activity was categorized according to WHO recommendations into three levels [1]:

- Low Physical Activity: <150 min/week of moderate activity. Includes a predominantly sedentary lifestyle with an energy expenditure of <1.5 METs (normal daily activities, slow walking, housework);
- Moderate Physical Activity: 150–300 min/week of moderate-intensity aerobic activity (e.g., brisk walking, cycling);
- Regular Physical Activity: >300 min/week or ≥150 min/week vigorous activity.

#### 2.3.2. Anthropometric Measurements

All measurements were performed once by a qualified rehabilitation specialist according to standardized protocols.

- Weight (kg): measured with a calibrated electronic scale (accuracy ±0.1 kg).
- Height (cm): measured with a portable height meter, with participants barefoot and standing in an anatomical position (accuracy ±0.1 cm).
- Waist circumference (WC): measured midway between the lower edge of the rib and the upper edge of the iliac crest, in a standing position. It is a criterion for central obesity, according to the International Diabetes Federation (IDF) from 2005 [18]. Increased risk is present in:
  ○ ≥94 cm for men
  ○ ≥80 cm for women
- Hip circumference (HC): measured at the widest part of the buttocks, at the level of the greater trochanters.

Based on these data, the following anthropometric indices were calculated: body mass index, waist-hip ratio and waist-height ratio.

- BMI: calculated as weight (kg)/height^2^ (m^2^). The categorization is according to the World Health Organization [1]:
  ○ <18.5 → underweight
  ○ 18.5–24.9 → normal weight
  ○ 25.0–29.9 → overweight
  ○ ≥30.0 → obesity
- Waist-to-hip ratio (WHR = WC/HC): indicator of adipose tissue distribution and predictor of metabolic disorders. According to WHO [15], an increased risk of cardiometabolic diseases is present at:
  ○ ≥0.90 in men
  ○ ≥0.85 in women
- Waist-to-height ratio (WHtR = WC/height): assessment of central obesity and cardiometabolic risk, with a threshold value ≥0.5 for both sexes [9,10]. The current recommendations of the National Institute for Health and Care Excellence (NICE, 2025) were also taken into account, according to which values of 0.50–0.59 indicate moderate, 0.60–0.69 high, and ≥0.70 very high risk of cardiometabolic diseases [19].

The combined use of BMI, WC, WHR, and WHtR enabled a comprehensive assessment of both general and central adiposity.

### 2.4. Statistical Analysis

Statistical analyses were performed using SPSS version 26 (IBM Corp., Armonk, NY, USA). Continuous variables were tested for normality (Shapiro–Wilk test) and are presented as mean ± standard deviation, while categorical variables are expressed as frequencies and percentages. Receiver operating characteristic (ROC) curve analysis was used to evaluate the discriminative ability of anthropometric indices for cardiometabolic outcomes. The area under the curve (AUC) was calculated and interpreted using standard thresholds. AUC values were interpreted as follows: 0.50–0.59 (poor), 0.60–0.69 (acceptable), 0.70–0.79 (good), 0.80–0.89 (very good), and ≥0.90 (excellent discrimination). Differences between AUCs were compared using DeLong’s test.

Multivariable logistic regression analyses were conducted using separate models for each anthropometric index to avoid multicollinearity. This was confirmed using the variance inflation factor (VIF), which indicated substantial overlap between certain indices. All models were stratified by sex and adjusted for age, smoking, physical activity, and sleep duration. Anthropometric indices as predictors of cardiometabolic outcomes were entered as standardized continuous variables (at 1 SD increments) to allow comparison of effect sizes. In this population, 1 SD corresponded to 5.5 kg/m^2^ for BMI, 13.6 cm for WC, 0.08 for WHR, and 0.08 for WHtR.

(1) Behavioral factors as predictors of cardiometabolic outcomes: These models included smoking, physical activity levels, and short sleep duration (<6 h), adjusted for age and anthropometric indices (BMI and WHR);
(2) Predictors of general obesity (BMI ≥25 kg/m^2^ and BMI ≥30 kg/m^2^): Adjusted for age, smoking, and short sleep duration. The phenomenon of quasi-complete separation occurred in the sex-specific models for general obesity (BMI ≥30), as there were zero or near-zero cases of obesity among participants reporting regular/high physical activity. In logistic regression, this distribution prevents the maximum likelihood estimation from converging, leading to disproportionately large odds ratios and unstable standard errors. To maintain the statistical integrity and stability of the multivariate models, physical activity was excluded from these specific iterations and analyzed separately;
(3) Predictors of central obesity (WC, WHR, and WHtR ≥0.5): Models were adjusted for age, smoking, physical inactivity, and short sleep duration. Adjusted odds ratios (aORs) with 95% confidence intervals (CIs) are reported.

Statistical significance was set at *p* < 0.05.

## 3. Results

### 3.1. Participant Characteristics

A total of 3512 participants were included, of whom 64.8% were women (n = 2276) and 35.2% were men (n = 1236). The mean age was 53.7 ± 14.9 years (range: 18–89), with no significant difference between sexes (*p* = 0.783).

The mean height was 165.0 ± 9.4 cm, mean weight 75.3 ± 15.7 kg, and mean BMI 27.6 ± 5.5 kg/m^2^. Mean waist circumference was 88.9 ± 13.6 cm, WHR 0.84 ± 0.08, and WHtR 0.54 ± 0.08 (Table 1).

Men were significantly taller (171.96 ± 8.5 vs. 161.29 ± 7.5) and heavier (79.83 ± 15.3 kg vs. 72.84 ± 15.4 kg) than women (*p* < 0.001). Mean BMI was higher in women (28.04 ± 5.8 kg/m^2^) than in men (26.99 ± 4.9 kg/m^2^). WHR was higher in men (0.89 vs. 0.83), while WHtR was slightly higher in women (0.54 vs. 0.53). Detailed characteristics are presented in Table 1.

### 3.2. Prevalence of General and Central Obesity

Overall, 68.4% of participants were classified as overweight or obese (BMI ≥25 kg/m^2^), and 29.6% had obesity (BMI ≥30 kg/m^2^). Elevated waist circumference was observed in 62.4% of participants, elevated WHR in 39.4%, and WHtR ≥0.5 in 66.9%. Women showed a higher prevalence of elevated waist circumference, while sex differences were less pronounced for WHtR (Table 2).

### 3.3. Cardiometabolic Risk Stratification

According to NICE criteria, 45.8% of participants were classified as having moderate risk, 21.0% as high risk, and 3.4% as very high cardiometabolic risk (Table 3).

Elevated cardiometabolic risk categories were also observed among individuals below the BMI-defined obesity threshold (Table 4).

### 3.4. Central Obesity in Individuals with Normal BMI

Central obesity was observed in approximately one-quarter of individuals with normal BMI (Table 5).

### 3.5. Age Patterns

The prevalence of both general and central obesity increased across age categories. The proportion of individuals with WHtR ≥ 0.5 increased from 43.1% in the youngest group (18–44 years) to 82.1% in the oldest group (75–89 years) (Table 6 and Table 7).

Significant differences in anthropometric indices were observed across age groups (*p* < 0.001). BMI increased with age, reaching the highest values in the 60–74 age group (28.82 ± 5.1 kg/m^2^), followed by a slight decrease in the oldest group (28.15 ± 4.9 kg/m^2^). In contrast, waist circumference and WHtR increased progressively across all age categories (Table 8).

### 3.6. Multicollinearity Assessment

Multicollinearity analysis showed high collinearity between waist circumference and WHtR, with variance inflation factor (VIF) values exceeding 10 (12.45 and 10.12, respectively). BMI (VIF = 3.84) and WHR (VIF = 1.58) showed lower levels of collinearity (Table 9).

Based on these findings, each anthropometric index was included in separate multivariable models.

### 3.7. Discrimination Ability of Anthropometric Indices

ROC analysis showed AUC values ranging from 0.70 to 0.85 across anthropometric indices (Table 10). BMI had the highest AUC for hypertension (0.852) and combined cardiometabolic outcomes (0.851). WHtR showed consistently high AUC values across outcomes, while WHR showed lower AUC values.

DeLong’s test indicated statistically significant differences between BMI and the other indices (Table 10).

### 3.8. Multivariable Models: Anthropometric Indices and Cardiometabolic Outcomes (Model Set 1)

All anthropometric indices were significantly associated with cardiometabolic outcomes (Table 11). BMI showed the strongest associations, followed by WHtR and waist circumference, while WHR showed lower effect sizes. In men, each 1 SD increase in BMI was associated with increased odds of combined cardiometabolic outcomes (aOR 2.88; 95% CI 2.42–3.43). WHtR showed consistent associations across both sexes.

### 3.9. Multivariable Models: Behavioral Factors and Cardiometabolic Outcomes (Model Set 2)

Behavioral factors showed sex-specific associations (Table 12). In women, short sleep duration (<6 h) was associated with higher odds of hypertension (aOR 2.09; 95% CI 1.35–3.25) and combined cardiometabolic outcomes (aOR 2.45). In men, smoking was associated with higher odds of diabetes (aOR 1.41) and combined cardiometabolic outcomes (aOR 1.59).

Regular physical activity was associated with lower odds of combined cardiometabolic outcomes in both sexes.

### 3.10. Multivariable Models: Predictors of General Obesity (Model Set 3)

Age was significantly associated with general obesity (Table 13). Each additional year increased the odds of obesity (aOR 1.05 in women; aOR 1.03 in men). Short sleep duration (<6 h) was associated with higher odds of obesity, particularly in women.

Due to quasi-complete separation, physical activity was excluded from these models.

### 3.11. Multivariable Models: Predictors of Central Obesity (Model Set 4)

Central obesity indices were associated with age and behavioral factors (Table 14).

In men, smoking was associated with higher WHtR (aOR 1.38). In women, short sleep duration was associated with higher WHR (aOR 2.54).

Low physical activity was associated with higher odds of central obesity in both sexes, with stronger associations observed in women.

## 4. Discussion

### 4.1. Main Findings

This study provides regional, quasi-representative data on the prevalence of general and central obesity and their cardiometabolic associations in Stara Zagora, Bulgaria. Due to its cross-sectional design, causal and temporal relationships cannot be established. Given the urban nature of the cohort, the findings are most applicable to similar urban adult populations rather than nationally representative samples.

A high prevalence of both general and central obesity was observed, with nearly 70% of participants classified as overweight or obese and almost one-third meeting criteria for obesity. These findings indicate a substantial burden of excess adiposity. Notably, central obesity indicators (WC, WHR, WHtR) identified a larger proportion of individuals at risk, suggesting that BMI alone may underestimate cardiometabolic risk.

Overall, 24.4% of participants were classified as having high or very high cardiometabolic risk according to NICE criteria. Importantly, elevated risk was also observed among individuals below the BMI-defined obesity threshold. This supports growing evidence that normal body weight does not necessarily reflect a metabolically healthy state [20,21,22,23,24,25,26,27,28].

### 4.2. Role of Central Obesity Indicators

Central obesity measures showed strong and consistent associations with cardiometabolic outcomes. WHtR demonstrated stable discriminatory ability across outcomes, while WC and WHR also provided meaningful contributions.

Although BMI showed the highest diagnostic accuracy in ROC analyses, central indices complemented its predictive value by capturing fat distribution rather than total body mass. This distinction is clinically relevant, as visceral adiposity is more strongly associated with metabolic dysfunction.

The presence of central obesity in approximately one-quarter of individuals with normal BMI further underscores the clinical relevance of these indices and supports their inclusion in routine risk assessment [29,30].

Differences in the performance of anthropometric measures suggest that they reflect distinct biological aspects of adiposity. The strong multicollinearity between WC and WHtR confirms their shared variance, whereas BMI and WHR appear to capture more independent components. This supports their interpretation as complementary rather than interchangeable measures.

### 4.3. Sex-Specific Differences

Clear sex differences were observed in both anthropometric characteristics and their associations with cardiometabolic risk. Women had a higher mean BMI and a higher prevalence of elevated waist circumference, whereas men exhibited higher WHR values. Despite the higher prevalence of abdominal obesity in women, associations between anthropometric indices and cardiometabolic outcomes were stronger in men, suggesting sex-specific differences in the metabolic impact of adiposity. This suggests sex-specific differences in the metabolic impact of adiposity and highlights the importance of sex-stratified analysis [31,32].

Behavioral factors also showed sex-specific patterns. In women, short sleep duration (<6 h) was a significant risk factor, increasing the odds of hypertension and combined cardiometabolic risk. This association may be explained by neuroendocrine dysregulation and increased sympathetic activity associated with sleep deprivation [33,34]. In contrast, smoking had a stronger association with metabolic risk in men.

Physical activity demonstrated a consistent protective effect in both sexes, highlighting its central role in prevention

### 4.4. Age-Related Patterns

Age-related variation was a key finding of this study. Both general and central obesity increased progressively with age, likely reflecting cumulative exposure to risk factors and age-related metabolic changes.

A divergence between BMI and central obesity indicators was observed in older age groups. While BMI plateaued or slightly declined in the oldest participants, WC and WHtR continued to increase. This suggests that BMI may underestimate adiposity in older adults due to age-related loss of muscle mass and fat redistribution [25,31].

In contrast, central obesity measures appear to retain their sensitivity across the lifespan. The increase in WHtR from 43.1% in younger adults to over 80% in the oldest group highlights the growing importance of abdominal adiposity with advancing age.

The interpretation of anthropometric indices also differs across age groups. In younger individuals, central obesity may indicate early metabolic imbalance. In middle-aged adults, elevated anthropometric measures more often coincide with established cardiometabolic disease. In older populations, interpretation is more complex due to physiological changes and comorbidities.

These findings support the need for age-specific interpretation rather than a uniform approach to obesity assessment.

### 4.5. Lifestyle Factors

Lifestyle factors were significantly associated with both obesity and cardiometabolic outcomes. Physical inactivity emerged as the strongest modifiable determinant, particularly in women. Although the high odds ratios should be interpreted cautiously due to the cross-sectional design and potential quasi-complete separation, the findings suggest a strong relationship between physical activity and fat distribution. The lower prevalence of central obesity among physically active individuals may indicate a potential protective threshold effect or “ceiling effect” of protection.

To further support the robustness of these findings, the association between physical inactivity and central obesity remained consistent across sex-stratified and age-adjusted models, suggesting that the observed effect is not solely driven by confounding. However, the magnitude of the odds ratios should be interpreted with caution, as quasi-complete separation may have led to overestimation of effect sizes in certain subgroups.

These findings are consistent with previous studies demonstrating a strong inverse association between physical activity and central adiposity [1], although effect sizes in the present analysis appear higher, likely reflecting the combined influence of behavioral clustering and the cross-sectional design.

Although formal sensitivity analyses were not performed, consistency across multiple adjusted and stratified models provides indirect support for the robustness of the findings.

Short sleep duration was identified as an important risk factor, particularly in women, while smoking showed stronger associations with metabolic risk in men. These findings are consistent with evidence that lifestyle behaviors—including physical activity, sleep, and smoking—play a key role in the development of central obesity and its metabolic consequences [35].

However, the results should be interpreted with caution due to the cross-sectional design and reliance on self-reported data.

### 4.6. Methodological Considerations in Interpretation

The use of multiple anthropometric indices enabled a comprehensive assessment of adiposity but required careful handling of multicollinearity. High VIF values for WC and WHtR justified their inclusion in separate models, ensuring model stability.

ROC analysis provided useful comparative insights; however, differences between indices should be interpreted as indicative rather than definitive.

### 4.7. Public Health Aspects

The findings highlight a substantial burden of general and central obesity and their strong association with cardiometabolic risk. The results support the use of combined anthropometric assessment, incorporating both BMI and central obesity indicators [36]. Age- and sex-specific differences further suggest that prevention strategies should be tailored rather than uniform. The consistent protective effect of physical activity underscores its central role in public health interventions.

Importantly, these results have direct implications for clinical practice and public health policy. The integration of waist-based measures, particularly waist-to-height ratio (WHtR), into routine primary care screening may improve early identification of individuals at increased cardiometabolic risk, including those with normal BMI. Given its simplicity and cost-effectiveness, WHtR can be easily implemented in large-scale screening and preventive programs without the need for specialized equipment.

From a public health perspective, the high prevalence of central obesity across all age groups highlights the need for targeted prevention strategies focusing on modifiable lifestyle factors, particularly physical activity and sleep. The observed age- and sex-specific patterns further support the development of tailored interventions rather than one-size-fits-all approaches. In addition, these results may inform regional health policies aimed at reducing the burden of cardiometabolic diseases in urban populations with similar demographic and behavioral characteristics.

Given the cross-sectional design and quasi-representative sample, the findings should be interpreted as population-specific associations rather than causal relationships. Longitudinal and nationally representative studies are needed to confirm these patterns.

### 4.8. Strengths and Limitations

This study has several strengths, including a large population-based sample and the use of multiple anthropometric indices to assess both general and central obesity. The combined application of ROC analysis and multivariable logistic regression enabled a comprehensive evaluation of both predictive performance and independent associations. By integrating prevalence data, adjusted associations, and diagnostic accuracy, the study provides clinically and public health–relevant insights into obesity-related risk stratification.

Several limitations should be acknowledged. First, the cross-sectional design precludes causal inference. Although multivariable models were used to adjust for potential confounders, the observed associations should be interpreted as indicative of relationships rather than causality. Longitudinal studies are needed to confirm temporal relationships.

Second, the non-random, convenience-based sampling approach may introduce selection bias and limit external validity. Individuals who are more health-conscious or have existing health conditions may have been more likely to participate. Therefore, the findings are most applicable to similar urban populations rather than nationally representative samples.

Third, behavioral variables were self-reported and may be subject to recall and reporting bias. The absence of objective measurement methods, such as accelerometry for physical activity or device-based sleep assessment, may have affected the accuracy of these variables.

Fourth, direct measures of body composition, such as bioelectrical impedance analysis (BIA) and dual-energy X-ray absorptiometry (DXA), were not available. Therefore, visceral adiposity could not be directly quantified.

Fifth, the study was conducted in a single urban region, which may further limit generalizability, as regional differences in socioeconomic status, occupational structure, and lifestyle patterns may influence both obesity prevalence and cardiometabolic risk profiles.

Finally, residual confounding cannot be excluded. Important variables such as dietary intake, socioeconomic status, and medication use were not included, which may have influenced the observed associations. In addition, the relatively high odds ratios observed for certain behavioral factors—particularly physical inactivity—should be interpreted with caution, as they may partly reflect residual confounding or quasi-complete separation in subgroup analyses.

## 5. Conclusions

In conclusion, this study demonstrates a high prevalence of both general (68.4%) and central obesity (66.9%) in the adult population of Stara Zagora, Bulgaria. The findings highlight that reliance on BMI alone may overlook significant cardiometabolic risk, particularly in the 24.4% of normal-weight individuals who exhibit high-risk central adiposity according to NICE criteria.

Our results further indicate that obesity is not a homogeneous condition but is influenced by sex-specific and behavioral factors, with short sleep duration emerging as a key predictor in women, while smoking shows stronger associations in men. The strong protective role of physical activity underscores its importance in prevention strategies.

Age-stratified analyses reveal a critical shift in anthropometric profiles across the lifespan. While BMI stabilizes or slightly declines in the oldest age group (75–89 years), central obesity indices, particularly WHtR, continue to increase. This suggests a progressive redistribution of adipose tissue toward the visceral compartment with aging. Consequently, WHtR appears to be a more stable and informative marker for metabolic risk assessment in older adults compared to BMI.

From a public health perspective, these findings support the integration of waist-based measures, such as WHtR, into routine clinical practice.

Given the quasi-representative nature of the sample, the results are primarily generalizable to similar urban populations and may inform targeted regional prevention strategies. Future longitudinal studies are needed to confirm these associations and to evaluate the long-term impact of sex-specific and behavioral interventions on cardiometabolic risk.

## Figures and Tables

**Table 1:** Baseline Characteristics of the Study Population.

Variable	Total (N = 3512)	Women (n = 2276)	Men (n = 1236)	*p*-Value
Age (years), mean ± SD	53.7 ± 14.9	53.66 ± 13.7	53.81 ± 16.8	0.783
Height (cm), mean ± SD	165.0 ± 9.4	161.29 ± 7.5	171.96 ± 8.5	<0.001
Weight (kg), mean ± SD	75.3 ± 15.7	72.84 ± 15.4	79.83 ± 15.3	<0.001
BMI (kg/m^2^), mean ± SD	27.6 ± 5.5	28.04 ± 5.8	26.99 ± 4.9	<0.001
WC (cm), mean ± SD	88.9 ± 13.6	87.6 ± 13.6	91.3 ± 13.2	<0.001
WHR, mean ± SD	0.84 ± 0.08	0.83 ± 0.08	0.89 ± 0.09	<0.001
WHtR, mean ± SD	0.54 ± 0.08	0.54 ± 0.09	0.53 ± 0.08	<0.001

BMI—body mass index; WC—waist circumference; WHR—waist-to-hip ratio; WHtR—waist-to-height ratio; SD—standard deviation.

**Table 2:** Prevalence of General and Central Obesity by Sex.

Variable	Total n (%)	Women n (%)	Men n (%)
BMI Categories			
Underweight	108 (3.1)	75 (3.0)	33 (2.9)
Normal weight	1000 (28.4)	673 (28.2)	327 (29.1)
Overweight	1364 (38.8)	929 (38.9)	435 (38.7)
Obesity	1040 (29.6)	712 (29.8)	328 (29.2)
Central Obesity Indicators			
Elevated WC	2192 (62.4)	1514 (63.4)	678 (60.4)
Elevated WHR	1384 (39.4)	956 (40.0)	428 (38.1)
WHtR ≥ 0.5	2348 (66.9)	1608 (67.3)	740 (65.9)

BMI—body mass index; WC—waist circumference; WHR—waist-to-hip ratio; WHtR—waist-to-height ratio. BMI categories: <18.5 underweight; 18.5–24.9 normal weight; 25.0–29.9 overweight; ≥30 obesity. Elevated WC: ≥94 cm (men), ≥80 cm (women); elevated WHR: >0.90 (men), >0.85 (women); WHtR ≥0.5.

**Table 3:** NICE Cardiometabolic Risk Classification.

Risk Category	n (%)
No risk	1048 (29.8)
Moderate risk	1608 (45.8)
High risk	736 (21.0)
Very high risk	120 (3.4)

WHtR- 0.50–0.59 indicates moderate, 0.60–0.69 high, and ≥0.70 very high risk of cardiometabolic diseases.

**Table 4:** Cardiometabolic Risk Categories Based on NICE Criteria.

BMI Category	Moderate Risk n (%)	High Risk n (%)	Very High Risk n (%)
Underweight	4 (3.7)	-	-
Normal weight	296 (29.6)	12 (1.2)	-
Overweight	984 (72.1)	140 (10.3)	140 (10.3)
Obesity	324 (31.2)	584 (56.2)	120 (11.5)

BMI < 18.5 → Underweight, 18.5–24.9 → Normal weight, 25.0–29.9 → Overweight, ≥30.0 → Obesity.

**Table 5:** Prevalence of Central Obesity among Underweight and Normal Weight Individuals.

Category	Elevated WC n (%)	Elevated WHR n (%)	Elevated WHtR n (%)
Underweight	4 (3.7)	16 (14.8)	-
Normal weight	256 (25.5)	260 (26.0)	248 (24.8)
Overweight	948 (69.5)	516 (37.8)	1068 (78.3)
Obesity	948 (94.6)	592 (56.9)	1032 (99.2)
Total	260	276	248

BMI < 18.5 → Underweight, 18.5–24.9 → Normal weight, Elevated waist circumference is defined as ≥94 cm in men and ≥80 cm in women. Elevated WHR >0.90 for men and >0.85 for women. Elevated WHtR ≥0.5 for both sexes.

**Table 6:** BMI Distribution by Age.

Age Category	Total N (%)	Normal Weight n (%)	Overweight n (%)	Obesity n (%)
Young Adults	808 (23)	340 (42.1)	260 (32.2)	164 (20.3)
Middle Age	1352 (38.5)	336 (24.9)	548 (40.5)	436 (32.2)
Elderly	1128 (32.1)	252 (22.3)	464 (41.1)	384 (34.0)
Senile	224 (6.4)	72 (32.1)	92 (41.1)	56 (25.0)
Total	3512	1000	1364	1040

Young adults—18–44; Middle Age 45–59; Elderly 60–74; Senile 75–89.

**Table 7:** Central Obesity & High/Very High Cardiometabolic Risk by Age.

Age Category	Total N (%)	Elevated WC n (%)	Elevated WHR n (%)	Elevated WHtR n (%)	High/Very High Risk by NICE n (%)
Young Adults	808 (23.0)	336 (41.6)	184 (22.8)	348 (43.1)	104 (12.9)
Middle Age	1352 (38.5)	872 (64.5)	440 (32.5)	948 (70.1)	292 (21.6)
Elderly	1128 (32.1)	816 (72.2)	612 (54.3)	868 (77.0)	368 (32.6)
Senile	224 (6.4)	168 (75.0)	148 (66.1)	184 (82.1)	92 (41.1)
Total	3512	2192	1384	2384	856

Young adults—18–44; Middle Age 45–59; Elderly 60–74; Senile 75–89.

**Table 8:** Mean Values of Anthropometric Indices across Age Categories.

Age Category	BMI Mean ± SD	WC Mean ± SD	WHtR Mean ± SD	WHR Mean ± SD	*p*-Value
Young Adults	24.31 ± 4.1	81.6 ± 11.2	0.48 ± 0.06	0.78 ± 0.07	< 0.001
Middle Age	27.54 ± 4.8	91.8 ± 12.5	0.56 ± 0.07	0.84 ± 0.08	< 0.001
Elderly	28.82 ± 5.1	98.4 ± 13.1	0.61 ± 0.08	0.88 ± 0.09	< 0.001
Senile	28.15 ± 4.9	101.2 ± 13.8	0.65 ± 0.09	0.91 ± 0.09	< 0.001

Young adults—18–44; Middle Age 45–59; Elderly 60–74; Senile 75–89.

**Table 9:** Multicollinearity Analysis of Anthropometric Indices.

Index	Variance Inflation Factor	Interpretation
BMI	3.84	Acceptable
WC	12.45	High collinearity
WHR	10.12	High collinearity
WHtR	1.58	Low collinearity

VIF values > 5.0 indicate significant multicollinearity. Due to the high overlap in variance explained by these parameters, each anthropometric predictor was evaluated in independent multivariable models to ensure the stability of the regression coefficients.

**Table 10:** Discriminative Ability of Anthropometric Indices and Statistical Comparison of AUCs.

Outcome	Index	AUC (95% CI)	ΔAUC vs. BMI	ΔAUC (95% CI)	*p*-Value (DeLong)
Diabetes	BMI	0.796 (0.754–0.838)	–	–	–
	WC	0.725 (0.709–0.740)	−0.071	(−0.088 to −0.054)	<0.001
	WHR	0.611 (0.594–0.631)	−0.185	(−0.210 to −0.160)	<0.001
	WHtR	0.741 (0.725–0.756)	−0.055	(−0.072 to −0.038)	<0.001
Hypertension	BMI	0.852 (0.839–0.864)	–	–	–
	WC	0.775 (0.759–0.790)	−0.077	(−0.091 to −0.063)	<0.001
	WHR	0.613 (0.593–0.632)	−0.239	(−0.265 to −0.213)	<0.001
	WHtR	0.799 (0.785–0.813)	−0.053	(−0.066 to −0.040)	<0.001
Diabetes + Hypertension	BMI	0.851 (0.838–0.865)	–	–	–
	WC	0.780 (0.763–0.795)	−0.071	(−0.086 to −0.056)	<0.001
	WHR	0.634 (0.613–0.654)	−0.217	(−0.244 to −0.190)	<0.001
	WHtR	0.812 (0.797–0.827)	−0.039	(−0.051 to −0.027)	<0.001

Area Under the Curve (AUC). BMI—body mass index; WC—waist circumference; WHR—waist-to-hip ratio; WHtR—waist-to-height ratio. We compare each index against BMI because it is the strongest predictor according to our data.

**Table 11:** Multivariable Logistic Regression for Anthropometric Indices and Cardiometabolic Outcomes (per 1 SD increase).

Outcome	Index	Men aOR (95% CI)	*p*-Value	Women aOR (95% CI)	*p*-Value
Diabetes	BMI	2.14 (1.82–2.52)	<0.001	1.92 (1.65–2.24)	<0.001
	WC	1.85 (1.56–2.19)	<0.001	1.74 (1.48–2.05)	<0.001
	WHR	1.42 (1.20–1.68)	<0.001	1.38 (1.18–1.62)	<0.001
	WHtR	1.90 (1.61–2.25)	<0.001	1.82 (1.55–2.14)	<0.001
Hypertension	BMI	2.56 (2.15–3.05)	<0.001	2.34 (2.02–2.71)	<0.001
	WC	2.10 (1.78–2.48)	<0.001	1.95 (1.68–2.26)	<0.001
	WHR	1.35 (1.15–1.58)	<0.001	1.41 (1.22–1.63)	<0.001
	WHtR	2.18 (1.85–2.57)	<0.001	2.05 (1.78–2.36)	<0.001
Diabetes + Hypertension	BMI	2.88 (2.42–3.43)	<0.001	2.65 (2.28–3.08)	<0.001
	WC	2.45 (2.06–2.92)	<0.001	2.18 (1.88–2.53)	<0.001
	WHR	1.52 (1.28–1.81)	<0.001	1.60 (1.38–1.86)	<0.001
	WHtR	2.52 (2.14–2.97)	<0.001	2.30 (1.98–2.67)	<0.001

OR—odds ratio; CI—confidence interval; BMI—body mass index; WC—waist circumference; WHR—waist-to-hip ratio; WHtR—waist-to-height ratio. All models were adjusted for age (continuous), smoking status, physical activity level, and sleep duration.

**Table 12:** Associations Between Behavioral Factors and Cardiometabolic Outcomes.

Outcome	Factor	Men aOR (95% CI)	*p*-Value	Women aOR (95% CI)	*p*-Value
Diabetes	Smoking	1.41 (1.21–1.64)	<0.001	1.12 (0.91–1.38)	0.285
	Moderate PA	0.75 (0.61–0.93)	0.008	0.82 (0.68–0.99)	0.042
	Regular PA	0.59 (0.44–0.79)	<0.001	0.64 (0.52–0.78)	<0.001
	Short sleep	1.22 (0.84–1.76)	0.300	1.45 (1.10–1.92)	0.008
Hypertension	Smoking	1.13 (0.95–1.33)	0.167	1.05 (0.88–1.25)	0.582
	Moderate PA	0.70 (0.56–0.87)	0.001	0.78 (0.65–0.94)	0.009
	Regular PA	0.60 (0.45–0.81)	<0.001	0.68 (0.55–0.84)	<0.001
	Short sleep	1.38 (1.05–1.82)	0.021	2.09 (1.35–3.25)	<0.001
Diabetes + Hypertension	Smoking	1.59 (1.33–1.90)	<0.001	1.21 (1.02–1.44)	0.028
	Moderate PA	0.73 (0.60–0.92)	<0.001	0.80 (0.66–0.97)	<0.001
	Regular PA	0.28 (0.17–0.44)	<0.001	0.35 (0.24–0.51)	<0.001
	Short sleep	1.71 (1.10–2.65)	0.015	2.45 (1.78–3.38)	<0.001

OR—odds ratio; CI—confidence interval; PA—physical activity; reference category: low physical activity: <150 min/week of moderate activity; Moderate PA: 150–300 min/week; Regular PA: >300 min/week or ≥150 min/week vigorous activity. Models adjusted for anthropometric indices, age, smoking status, physical activity level, and sleep duration.

**Table 13:** Multivariable Logistic Regression for General Obesity.

Predictor	BMI ≥25 aOR (95% CI)	*p*-Value	BMI ≥30 aOR (95% CI)	*p*-Value
Men				
Age (per year)	1.02 (1.01–1.03)	0.045	0.98 (0.97–0.99)	0.002
Smoking	0.82 (0.65–1.03)	0.072	2.58 (1.87–3.55)	<0.001
Short sleep (<6 h)	0.20 (0.11–0.36)	<0.001	1.72 (0.86–3.42)	0.120
Low physical activity	1.13 (0.87–1.45)	0.002	*	<0.001
Women				
Age (per year)	1.00 (0.99–1.01)	0.820	0.97 (0.96–0.98)	<0.001
Smoking	1.04 (0.87–1.23)	0.450	1.12 (0.91–1.39)	0.150
Short sleep (<6 h)	0.27 (0.17–0.45)	<0.001	2.98 (1.89–4.67)	<0.001
Low physical activity	1.33 (1.10–1.60)	<0.003	*	<0.001

* Variable excluded from the final model due to quasi-complete separation (zero cases in the active/obese cell).

**Table 14:** Multivariable Predictors of Central Obesity.

Predictor	Elevated WC aOR (95% CI)	*p*-Value	Elevated WHR aOR (95% CI)	*p*-Value	WHtR ≥0.5 aOR (95% CI)	*p*-Value
Men						
Age (per year)	1.02 (1.01–1.03)	<0.001	1.03 (1.02–1.04)	<0.001	1.03 (1.02–1.04)	<0.001
Smoking	1.28 (1.12–1.55)	0.051	1.25 (1.25–1.68)	0.061	1.38 (1.15–1.58)	0.020
Low physical activity	5.33 (4.06–7.00)	<0.001	1.61 (1.25–2.07)	<0.001	6.33 (4.75–8.44)	<0.001
Short sleep (<6 h)	0.57 (0.32–0.99)	0.048	0.83 (0.95–1.65)	0.509	0.42 (0.22–0.78)	0.006
Women						
Age (per year)	1.01 (1.00–1.02)	<0.001	1.03 (1.02–1.04)	<0.001	1.02 (1.01–1.03)	<0.001
Smoking	1.09 (0.88–1.35)	0.413	1.13 (0.94–1.36)	0.168	1.11 (0.90–1.38)	0.310
Low physical activity	8.06 (6.40–10.15)	<0.001	2.37 (1.93–2.91)	<0.001	9.27 (7.42–11.59)	<0.001
Short sleep (<6 h)	1.18 (0.62–2.26)	0.610	2.54 (1.63–3.95)	<0.001	1.26 (0.65–2.41)	0.484

Elevated waist circumference is defined as ≥94 cm in men and ≥80 cm in women. Elevated WHR >0.90 for men and >0.85 for women. Elevated WHtR ≥0.5 for both sexes.

## Data Availability

The data presented in this study are available on request from the corresponding author due to privacy and ethical restrictions.

## Abbreviations

The following abbreviations are used in this manuscript:

AUC	Area under the curve
BMI	Body mass index
IDF	The International Diabetes Federation
NICE	National Institute for Health and Care Excellence
PA	Physical activity
ROC	Receiver operating characteristic
VIF	Variance inflation factor
WHR	Waist-to-hip-ratio
WHtR	Waist-to-height-ratio
WC	Waist circumference
WHO	World Health Organization
